# Distorted chemosensory perception and female sex associate with persistent smell and/or taste loss in people with SARS-CoV-2 antibodies: a community based cohort study investigating clinical course and resolution of acute smell and/or taste loss in people with and without SARS-CoV-2 antibodies in London, UK

**DOI:** 10.1186/s12879-021-05927-w

**Published:** 2021-02-25

**Authors:** Janine Makaronidis, Chloe Firman, Cormac G. Magee, Jessica Mok, Nyaladzi Balogun, Matt Lechner, Alisia Carnemolla, Rachel L. Batterham

**Affiliations:** 1 UCL Centre for Obesity Research, Division of Medicine, University College London, London, UK; 2 Bariatric Centre for Weight Management and Metabolic Surgery, University College London Hospital, London, UK; 3 National Institute of Health Research, UCLH Biomedical Research Centre, London, UK; 4 UCL Cancer Institute, University College London, London, UK; 5 Department of Otolaryngology-Head and Neck Surgery, Stanford University School of Medicine, Palo Alto, CA USA; 6 Barts Health NHS Trust, London, UK

**Keywords:** COVID-19, Long COVID-19, SARS-CoV-2 IgG/IgM, Smell loss, Taste loss, Smell recovery, Taste recovery

## Abstract

**Background:**

Loss of smell and/or taste are cardinal symptoms of COVID-19. ‘Long-COVID’, persistence of symptoms, affects around one fifth of people. However, data regarding the clinical resolution of loss of smell and/or taste are lacking. In this study we assess smell and taste loss resolution at 4–6 week follow-up, aim to identify risk factors for persistent smell loss and describe smell loss as a feature of long-COVID in a community cohort in London with known SARS-CoV-2 IgG/IgM antibody status. We also compare subjective and objective smell assessments in a subset of participants.

**Methods:**

Four hundred sixty-seven participants with acute loss of smell and/or taste who had undergone SARS-CoV-2 IgG/IgM antibody testing 4–6 weeks earlier completed a follow-up questionnaire about resolution of their symptoms. A subsample of 50 participants completed an objective olfactory test and results were compared to subjective smell evaluations.

**Results:**

People with SARS-CoV-2 antibodies with an acute loss of sense of smell and taste were significantly less likely to recover their sense of smell/taste than people who were seronegative (smell recovery: 57.7% vs. 72.1%, *p* = 0.027. taste recovery 66.2% vs. 80.3%, *p* = 0.017). In SARS-CoV-2 positive participants, a higher percentage of male participants reported full resolution of smell loss (72.8% vs. 51.4%; *p* < 0.001) compared to female participants, who were almost 2.5-times more likely to have ongoing smell loss after 4–6 weeks (OR 2.46, 95%CI 1.47–4.13, *p* = 0.001). Female participants with SARS-CoV-2 antibodies and unresolved smell loss and unresolved taste loss were significantly older (> 40 years) than those who reported full resolution. Participants who experienced parosmia reported lower smell recovery rates and participants with distorted taste perception lower taste recovery rates. Parosmia had a significant association to unresolved smell loss (OR 2.47, 95%CI 1.54–4.00, *p* < 0.001).

**Conclusion:**

Although smell and/or taste loss are often transient manifestations of COVID-19, 42% of participants had ongoing loss of smell, 34% loss of taste and 36% loss of smell and taste at 4–6 weeks follow-up, which constitute symptoms of ‘long-COVID’. Females (particularly > 40 years) and people with a distorted perception of their sense of smell/taste are likely to benefit from prioritised early therapeutic interventions.

**Trials registration:**

ClinicalTrials.govNCT04377815 Date of registration: 23/04/2020.

**Supplementary Information:**

The online version contains supplementary material available at 10.1186/s12879-021-05927-w.

## Introduction

Severe Acute Respiratory Syndrome Coronavirus 2 (SARS-CoV-2), which results in coronavirus disease 2019 (COVID-19), emerged in 2019 resulting in a global pandemic with over 85 million cases and 1.8 million deaths reported worldwide [1]. The association between COVID-19 and smell and taste loss was established in March 2020 and has since been identified among the most specific symptoms of COVID-19, with implications for case identification, isolation and tracing [2, 3]. We previously reported seroprevalence of SARS-CoV-2 IgG/IgM antibodies of 78% in a community cohort in London, UK who developed an acute loss of their sense of smell and/or taste during the peak of the first local wave of the pandemic [4]. We also showed that seropositivity for SARS-CoV-2 antibodies was three times more likely for people with smell loss compared to taste loss.

With the ongoing spread of SARS-CoV-2 globally, COVID-19 and its complications are continuing to affect millions globally. Despite the development of vaccinations resulting in optimism that the pandemic will be contained, healthcare providers worldwide will continue to face the challenge of an unprecedented incidence of COVID-19 related morbidity. It is now evident that symptoms can persist beyond resolution of the acute systemic viral infection and cause a chronic condition, termed ‘long-COVID’, in up to a quarter of cases [5]. ‘Long covid’ (> 4 weeks) as well as ‘post-acute covid’ (> 3 weeks) and ‘chronic covid’ (> 12 weeks) have been used to describe ongoing symptoms of the disease [6, 7]. Symptoms persisting longer than 5 weeks are estimated to occur in 1 in 5 and symptoms and lasting longer than 12 weeks in 1 in 10 people, according to data published by the UK Office for National Statistics (ONS) [8]. Identifying the people most at risk of long-term symptoms will be key to guide monitoring in order to deliver follow-up care and therapeutic interventions to patients with long COVID-19 disease.

Despite the recognition of loss of smell and/or taste as key symptoms of COVID-19 there remains a paucity of data within the literature regarding the clinical course, recovery rates and demographic risk factors for long-lasting symptoms. Post-viral smell loss from other respiratory pathogens is typically a short-lived phenomenon and recovery usually coincides with resolution of other viral symptoms [9, 10]. In contrast, current data from COVID-19 patients, suggest a wide range of recovery times, from a few days to several months [11–13]. This observation raises concerns of unresolved smell loss as a manifestation of long-COVID. Therefore, identifying risk factors for persistent smell loss will be key to guide therapeutic interventions, such as olfactory rehabilitation and use of corticosteroids, once these are widely available [14, 15]. Furthermore, given the negative impact of these symptoms on quality of life and safety, research on prognostic information is warranted to inform patients, their carers, as well as treatment algorithms [16]. In this follow-up study, we aim to describe and compare the temporal resolution patterns of loss of smell and/or taste in a community cohort with acute smell and/or taste loss with and without SARS-CoV-2 antibodies. This study will focus on smell loss resolution and aim to identify risk factors for persistent smell loss, as well as describe smell loss as a feature of long-COVID. Finally, to address the original study’s limitations of reliance on self-reported symptoms, we will correlate self-reported smell function with objective olfactory testing.

## Methods

The study was conducted in London, UK. Participants were recruited between 23 April and 14 May 2020, during the local first wave of the pandemic, prior to recognition of smell and taste loss as symptoms of COVID-19. Primary care centres sent out text messages to invite people with an acute loss of their sense of smell and/or taste to participate.. Participants were recruited via an online platform, as previously described [4].

Inclusion criteria were age of 18 years and above, being proficient in written and spoken English and having access to a device that can perform video calls. Participants who experienced a loss of the sense of smell or taste with a duration greater than 1 month were excluded [4]. Participation was voluntary and written informed consent was obtained electronically. Participants were enrolled through consecutive recruitment of eligible participants, who completed an online questionnaire (see Additional File 1). The baseline questionnaire included questions on participants’ demographics and their symptoms [4].

SARS-CoV-2 immunoglobulin G (IgG)/ immunoglobulin M (IgM) antibody testing was carried out via a telemedicine consultation as previously described [4], scheduled taking into consideration the participants’ date of symptom onset. The test used was a lateral flow immunoassay detecting IgM and IgG antibodies to SARS-CoV-2 (Wuhan UNscience Biotechnology Co., Ltd. COVID-19 Antibody IgM/IgG) and has a relative sensitivity of 98.8% (95% CI 97.3–99.6%) and a relative specificity of 98.0% (95% CI 97.15–98.7%) [17]. A link to a follow-up questionnaire was sent to participants’ registered email addresses 4 weeks after they completed their original questionnaire. The follow up questionnaire (see Additional File 1) contained questions about resolution of their smell and taste loss, as well as resolution of the other symptoms of COVID-19 and admission to hospital. Reminders were sent to participants who did not complete the questionnaire at 72 h and 7 days and the platform remained open for questionnaire completion for a further 4 weeks (22 May to 20 July 2020).

In order to correlate participants’ questionnaire responses about their loss of smell with objective olfactory testing, a subsample of 50 participants were recruited for objective smell testing. Participants received a separate information sheet and informed consent was obtained electronically using a separate consent form. The University of Pennsylvania Smell Identification Test (UPSIT), a 40-item smell test which is validated to be self-administered, was used [18]. UPSIT kits were sent to recruited participants together with instructions on completing the test. In view of the time elapsed between the completion of the follow-up questionnaire and the UPSIT testing, prior to testing, participants were asked how they subjectively perceived their smell function. Photographs of the booklet were obtained and the tests were scored by a healthcare professional, who then explained the results to participants. Participants’ subjective smell function was correlated with their UPSIT results.

Ethical approval for this study was issued by the National Health Service Queen’s Square Research Ethics Committee (IRAS Project ID 282668, ClinicalTrials.gov: NCT04377815). The study was conducted in line with the declaration of Helsinki and Good Clinical Practice.

### Statistical analysis

The recruitment target for this study was set at 500 participants. A sample size calculation yielded a sample size of 385, which including a 15% attrition rate was initially increased to 453 participants and then increased to 500 to allow for larger attrition [4]. The recruitment target was exceeded to increase accuracy.

Data analysis was performed using GraphPad Prism version 8 (https://www.graphpad.com/scientific-software/prism/) and SPSS version 26 (https://www.ibm.com/uk-en/products/spss-statistics).

Descriptive statistics were performed; means (plus standard deviation [SD]) were calculated for continuous variables and numbers (*n*, with percentages) for categorical variables. Categorical data were analysed using chi-squared tests. The significance level for multiple comparisons was adjusted using a Bonferroni correction. Parametric and non-parametric tests were used as appropriate to analyse continuous data. Logistic regression analysis was performed to estimate the association between smell loss resolution and additional factors including participant’s age, sex, ethnicity, smoking status and smell loss pattern. A Spearman Rank correlation analysis was performed to investigate the association between participants’ perceived smell function and an objective assessment of their olfactory function.

## Results

### Study population

A total of 467 out of the 569 participants who enrolled and underwent a SARS-CoV-2 antibody test completed the follow-up questionnaire, yielding a follow-up rate of 82.1%. The demographics of the entire study cohort can be seen in Additional File 2. Out of the cohort of 467 who completed the follow-up questionnaire, participants with positive and negative SARS-CoV-2 antibodies were comparable in terms of age, gender, smoking status and ethnicity (Table 1). Admission to hospital was reported by 1.6% (*n* = 7) of participants in the antibody positive group vs. 3.1% (*n* = 4) in the antibody negative group (*p* = 0.097).

**Table 1:** Demographics of participants followed up after 4–6 weeks with positive and negative SARS-CoV-2 antibodies

Demographics	SARS-CoV-2 IgG/IgM positive (***n*** = 381)	SARS-CoV-2 IgG/IgM negative (***n*** = 86)	***p***-value (0.05)
**Gender**			
Female	70.9% (*n* = 270)	66.3% (*n* = 57)	*0.382*
Male	28.8% (*n* = 110)	33.7% (*n* = 29)	
Other	0.3% (*n* = 1)	0	*0.450*
**Age (years)**	39.67 ± 12.12	40.25 ± 12.33	*0.689*
**Ethnicity** ^**a**^			
White	83.7% (*n* = 319)	81.4% (*n* = 70)	*0.600*
Mixed/Multiple Ethnicities	5.5% (*n* = 21)	4.7% (*n* = 4)	*0.749*
Asian/Asian British	5% (*n* = 19)	4.7% (*n* = 4)	*0.897*
Black/African/Caribbean/Black British	1.6% (*n* = 6)	2.3% (*n* = 2)	*0.628*
‘Other’	3.7% (*n* = 14)	3.5% (*n* = 3)	*0.933*
**Smoking status**			
Current/ Ex-smoker	42% (*n* = 160)	44.2% (*n* = 38)	*0.710*
Never smoked	58% (*n* = 221)	55.8% (*n* = 48)	

Figures presented as % with total number (n). SARS-CoV-2, severe acute respiratory syndrome coronavirus 2.

^**a**^5 participants opted not to disclose their ethnicity

### Smell and taste loss and resolution at 4–6 weeks follow-up

The frequency of reported smell and/or taste loss at baseline in participants who completed the follow-up questionnaire can be seen in Table 2.

**Table 2:** Loss of smell and/or taste in SARS-CoV-2 IgG/IgM positive and negative participants

	SARS-CoV-2 IgG/IgM positive (*n* = 381)	SARS-CoV-2 IgG/IgM negative (*n* = 86)	*p*-value (0.05)
**Sense of smell**			
**Loss of sense of smell (complete and partial)**	93.7% (*n* = 358)	79.1% (*n* = 68)	***< 0.001***
Partial loss of smell	25.7% (*n* = 92)	47.1% (*n* = 32)	***< 0.001***
Complete loss of smell	74.3% (*n* = 266)	52.9% (*n* = 36)	
**Parosmia** (distorted sense of smell)	29.7% (*n* = 113)	22.1% (*n* = 19)	*0.159*
**Sense of taste**			
**Loss of sense of taste (complete and partial)**	89.8% (*n* = 342)	88.4% (*n* = 76)	*0.704*
Partial loss of taste	54.1% (*n* = 185)	69.7% (*n* = 53)	***0.013***
Complete loss of taste	45.9% (*n* = 157)	30.3% (*n* = 23)	
**Dysgeusia** (distorted sense of taste)	43.8% (*n* = 167)	36% (n = 31)	*0.187*
**Experience of taste without eating/drinking**	19.9% (n = 76)	23.3% (*n* = 20)	*0.492*
**Only loss of smell**	10% (*n* = 38)	11.6% (*n* = 10)	*0.649*
**Only loss of taste**	6.3% (*n* = 24)	20.9% (*n* = 18)	***< 0.001***
**Loss of sense of smell and taste (partial and complete)**	83.7% (*n* = 319)	67.4% (*n* = 58)	***< 0.001***

Out of 467 patients followed up at 4–6 weeks, 57.7% (*n* = 206) of participants with positive SARS-CoV-2 antibodies reported full resolution of their smell loss, compared to 72.1% (*n* = 49) of participants with a negative antibody test (*p* = 0.027). Out of the participants with positive SARS-CoV-2 antibodies, 38.4% (*n* = 137) reported partial and 3.9% (*n* = 14) reported no resolution of their smell loss at the time of follow-up. Out of participants with negative SARS-CoV-2 antibodies 25.0% (*n* = 17) reported partial and 2.9% (*n* = 2) no resolution of their smell loss.

Full resolution of taste loss was reported by 66.2% (*n* = 227) of participants with SARS-CoV-2 antibodies and 80.3% (*n* = 61) of participants with negative SARS-CoV-2 antibodies (*p* = 0.017). Out of participants with positive SARS-CoV-2 antibodies, 31.2% (*n* = 107) reported partial and 2.6% (*n* = 9) no resolution of their taste loss at the time of follow up. Out of participants with negative SARS-CoV-2 antibodies, 19.7% (*n* = 15) reported partial resolution of their taste loss (0 participants reported ‘no resolution’ of taste loss). Out of participants with positive SARS-CoV-2 antibodies, only 24 experienced a loss of their sense of taste in the absence of a loss of smell. The demographics were comparable to those of the entire SARS-CoV-2 positive cohort, with a mean age of 38.4 ± 14.21 years and 70.8% (*n* = 17) of participants of female sex.

For subsequent analyses participants with partial and no resolution were grouped together in order to enable comparison between participants who achieved full resolution versus those who had ongoing smell and/or taste impairment at the time of follow-up. Table 3 shows results regarding resolution vs. no resolution of smell loss, taste loss and combined smell/taste loss in participants with positive and negative SARS-CoV-2 antibodies. A higher percentage of participants without SARS-CoV-2 antibodies fully recovered their sense of smell (72.1% vs 57.7%. *p* = 0.027), their sense of taste (80.3% vs 66.2%, *p* = 0.017) and both their senses of smell and taste (79.6% vs 64.0%, *p* = 0.026).

**Table 3:** Smell and/or taste loss resolution in SARS-CoV-2 IgG/IgM positive and negative participants

Pattern of resolution	SARS-CoV-2 IgG/IgM positive	SARS-CoV-2 IgG/IgM negative	***p***-Value (0.05)
**Smell loss**	**Total (*n*** **= 357)**	**Total (*n*** **= 68)**	
No/partial resolution	42.3% (*n* = 151)	27.9% (*n* = 19)	***0.027***
Full resolution	57.7% (*n* = 206)	72.1% (*n* = 49)	
**Taste loss**	**Total (*n*** **= 343)**	**Total (*n*** **= 76)**	
No/partial resolution	33.8% (*n* = 116)	19.7% (*n* = 15)	***0.017***
Full resolution	66.2% (*n* = 227)	(80.3%) (*n* = 61)	
**Combined smell and taste loss**	**Total (*n*** **= 261)**	**Total (*n*** **= 54)**	
No/partial resolution	36.0% (*n* = 94)	20.4% (*n* = 11)	***0.026***
Full resolution	64.0% (*n* = 167)	79.6% (*n* = 43)	

In participants with SARS-CoV-2 antibodies who reported full resolution of their smell loss, a full recovery of the sense of smell was reported to have occurred within 1 week in 11.7%, within 1–2 weeks in 26%, within 2–4 weeks in 26.5% and within > 4 weeks in 35.8%.

### The effects of smell loss pattern and presence of parosmia on recovery of the sense of smell in participants with SARS-CoV-2 IgG/IgM antibodies

The effect of complete vs. partial smell loss and the presence of parosmia on smell recovery in participants with positive SARS-CoV-2 antibodies was investigated. In participants who experienced complete loss of their sense of smell, full sense of smell recovery was reported by 54.5% compared to 67.4% in participants who reported a partial loss of their sense of smell (54.5% vs 67.4%, *p* = 0.032).

Out of participants who reported parosmia at the time of their smell loss, full recovery was reported by 41.4% compared to 65% in participants with smell loss who did not experience parosmia (41.4% vs. 65%, *p* < 0.001).

### The effects of taste loss pattern and presence of dysgeusia on recovery of the sense of taste in participants with SARS-CoV-2 IgG/IgM antibodies

The effect of complete vs. partial taste loss and the presence of dysgeusia on smell recovery in participants with positive SARS-CoV-2 antibodies was investigated. There was no significant difference in the reported rates of recovery of taste loss in participant who reported complete vs. partial loss of their sense of taste (64.7% vs. 67.9%, *p* = 0.525).

Out of participants who experienced dysgeusia at time of their loss of taste, a significantly lower proportion reported full resolution of their taste loss, compared to participants who did not experience dysgeusia (60.7% vs. 71.4%, *p* = 0.036).

Participants who experienced taste sensations in the absence of eating or drinking reported lower resolution rates compared to participants who did not (51.2% vs 71.0%, *p* = 0.001).

### The effect of sex and age on the recovery of the sense of smell and taste in participants with SARS-CoV-2 IgG/IgM antibodies

Full recovery of sense of smell was more prevalent among males compared to females (72.8% in males vs. 51.4% in females, *p* < 0.001). Similarly, full taste loss resolution was more common in males vs. females (80.8% vs. 60.1%, *p* < 0.001) as was full resolution of combined smell/taste loss (69.6% vs. 4.1%, *p* < 0.001); Table 4.

**Table 4:** Resolution of loss of smell, loss of taste and combined loss of smell and taste in female vs. male participants with SARS-CoV-2 IgG/IgM antibodies

	Female (***N*** = 270)	Male (***N*** = 110)	***p***-value (0.05)
**Smell loss resolution**			
**Full resolution**	51.4% (*n* = 130)	72.8% (*n* = 75)	***< 0.001***
**No/partial resolution**	48.6% (*n* = 123)	27.1% (*n* = 28)	
**Taste loss resolution**			
**Full resolution**	60.1% (*n* = 146)	80.8% (*n* = 80)	***< 0.001***
**No/partial resolution**	39.9% (*n* = 97)	19.2% (n = 19)	
**Combined smell and taste loss resolution**			
**Full resolution**	45.1% (*n* = 102)	69.6% (*n* = 64)	***< 0.001***
**No/partial resolution**	54.9% (*n* = 124)	30.4% (n = 28)	

The effect of age on resolution of smell and taste loss was evaluated. Mean age of male and female participants was comparable for participants who experienced full resolution of the loss in their sense of smell (40.4 ± 13.2 years in males vs. 38.1 ± 11.3 in females, *p* = 0.333), their sense of taste (40.3 ± 13.4 vs .37.1 ± 10.7 *p* = 0.153) and combined loss of smell and taste (40.0 ± 12.6 vs. 37.1 ± 11.0, *p* = 0.122). In participants with loss of their sense of taste that did not resolve at the time of follow-up, mean age was significantly higher in females compared to males (42.7 ± 12.5 years vs. 37.6 ± 12.6 years, *p* = 0.030). Mean age was also significantly higher in female participants with unresolved combined loss of smell and taste loss compared to male participants (42.8 ± 12.5 vs. 34.6 ± 10.4, *p* = 0.001). Mean age of female participants with unresolved smell loss was 41.6 ± 11.7 years compared to 37.4 ± 12.7 years in male participants, however this borderline difference did not reach statistical significance (*p* = 0.053).

In light of the above findings, we further evaluated the effect of age on smell and/or taste loss resolution in female participants. A significantly higher age was observed in female participants without resolution compared to those with full resolution of the loss of their sense of smell (41.6 ± 11.7 yrs. vs. 38.1 ± 11.3 yrs., *p* = 0.010), their sense of taste (42.7 ± 12.5 yrs. vs. 37.1 ± 10.7 yrs., *p* < 0.001) and combined smell and taste (42.8 ± 12.5 yrs. vs. 37.1 ± 11.0 yrs., *p* < 0.001).

### Predictors of persisting smell loss in a community population with SARS-CoV-2 antibodies and acute loss of their sense of smell

Logistic regression was used to explore the relative importance of participant’s age, sex, ethnicity, smoking status, presence of parosmia and smell loss pattern as risk factors for persistent smell loss at > 4 weeks from onset.

Female participants were almost 2.5 times more likely to have ongoing smell loss after 4 weeks compared to participants of male sex (OR 2.46, 95% CI 1.47 to 4.13, *p* = 0.001). Parosmia was also shown to have a significant association with unresolved smell loss at 4–6 week follow-up (OR 2.47, 95%CI 1.54 to 4.00, *p* < 0.001), in a model adjusting for the age, ethnicity, patterns of smell loss (complete vs partial) and smoking; Table 5.

**Table 5:** Logistic regression exploring the association between age, sex, ethnicity, smoking status, presence of parosmia and smell loss pattern (complete vs partial) and no resolution of smell loss at 4 weeks follow up

Variable	B	OR	95% CI (lower)	95% CI (upper)	***p*** value
**Age**	0.13	1.013	.994	1.032	*0.172*
**Ethnicity**	0.96	1.101	.595	2.034	*0.760*
**Complete anosmia**	0.529	1.697	0.998	2.884	*0.051*
**Parosmia**	0.904	2.470	1.539	3.966	***< 0.001***
**Sex (Female)**	0.901	2.461	1.468	4.126	***0.001***
**Smoking**	0.303	1.355	0.604	3.038	*0.462*

### Persistent smell and/or taste loss as a manifestation of long COVID

At the end of the 4–6 week follow-up period 42.3% (*n* = 151) of participants with positive SARS-CoV-2 antibodies had ongoing smell loss, 33.8% experienced ongoing taste loss and 36% had ongoing taste and smell loss. We also evaluated the resolution of other symptoms of COVID-19 at the end of the follow-up period in participants positive for SARS-CoV-2 antibodies. Out of 134 participants with unresolved smell loss who reported additional COVID-19 symptoms on their original questionnaire, 29.1% (*n* = 39) had at least 1 additional unresolved symptom at the time they completed their follow-up questionnaire, compared to 19.9% (*n* = 35) of participants with full resolution of their smell loss (29.1% vs 19.9%, *p* = 0.059). The most commonly reported unresolved symptoms were shortness of breath, chest pain and muscle/joint pains.

### Objective smell testing in a subsample of participants and correlation with perceived smell function

A subsample of 50 participants underwent objective olfactory testing using the UPSIT. 84% were female (*n* = 42) and 16% (*n* = 8) male. The mean age was 39.6 ± 13.5 years and mean duration of test date from the onset of symptoms was 21.6 ± 4.7 weeks. 76% (*n* = 38) of participants had complete loss of their sense of smell at the time of the original questionnaire and 24% (*n* = 12) partial loss of smell. At the time of the follow-up questionnaire 16% (*n* = 8) reported their smell loss ‘did not resolve’, 42% (*n* = 21) reported their smell loss ‘resolved partially’ and 42% (*n* = 21) reported their smell loss ‘resolved fully’.

In view of the time elapsed between the completion of the follow-up questionnaire and the UPSIT testing, prior to testing, participants were asked how they subjectively perceived their smell function at the time of the UPSIT test. Their answers were grouped into: ‘No or minimal sense of smell’, ‘Sense of smell improved but not fully recovered’ or ‘Sense of smell fully recovered’. The mean UPSIT test score was 29.1 ± 7.5 points. UPSIT testing revealed total anosmia in 5 participants (10%), severe microsmia in 5 (10%), moderate microsmia in 8 (16%), mild microsmia in 6 (12%) and normosmia (normal smell function) in 26 (52%).

Table 6 illustrates a comparison of participants’ perceived smell function and their UPSIT test result, by test result category. A Spearman rank correlation analysis found a significant correlation between perceived smell function and UPSIT test result category (*r* = 0.84 ± 0.71 to 0.90, *p* < 0.001).

**Table 6:** Comparison between UPSIT test result and perceived smell function in a study subgroup of 50 participants

	Minimal/no sense of smell (***n*** = 11)	Improved sense of smell, not fully recovered (***n*** = 9)	Fully recovered (***n*** = 30)
Total anosmia	45.5% (5)	0%	0%
Severe microsmia	45.5% (5)	0%	0%
Moderate microsmia	9.0% (1)	77.8% (7)	0%
Mild microsmia	0%	11.1% (1)	16.7% (n = 5)
Normosmia	0%	11.1% (1)	83.3% (*n* = 25)
**Spearman r**	**0.95**	**−0.63**	**−0.89**

## Discussion

We report longitudinal data from a community cohort with a new loss in their sense of smell and/or taste and resolution of these symptoms both in people with positive and negative SARS-CoV-2 IgG/IgM antibodies. Our data come from an entirely community based cohort with a low hospital admission rate where loss of taste and/or smell are the predominant symptoms. 77.9% of our cohort had positive SARS-CoV-2 antibodies and the study had a follow-up completion rate of 82.2%. We report a higher rate of recovery of smell loss (72.1% vs. 57.7%; *p* = 0.027), taste loss (80.3% vs. 66.2%; *p* = 0.017) and combined smell and taste loss (79.6% vs. 64%; *p* = 0.026) in participants who tested negative compared to participants who tested positive for SARS-CoV-2 antibodies. Participants in this study were tested for antibodies following recruitment and completing the baseline questionnaire, taking into consideration their onset of symptoms. Evidence into persistence of these antibiodes now suggests that these persist for several months, even in populations with mild disease [19, 20].

Importantly, our study highlights the high percentage of patients with ongoing smell loss (42.3%), ongoing taste loss (33.8%) and combined smell and taste loss (36.0%). The observed smell loss resolution rate of 57.7% in participants with SARS-CoV-2 antibodies within 4–6 weeks in our study is in line with existing literature [12, 21]. Dell’Era et al. similarly reported that in 355 participants with COVID-19, 70% reported either smell loss and/or taste loss during infection [22]. 49.5% of participants reported full resolution of both sense of smell/taste after 14 days since the onset of symptoms, increasing to 62.9% at time of interview (23 days median, range 15–31), with a median recovery time of 10 days. In contrast to our community-based study, their findings come from a hospitalised patient cohort. Resolution rates in the literature currently range from 29 to 92.8% [11, 12, 23]. Discrepancies are likely due to differences in study populations, sample size, location and duration of follow up since onset of symptoms.

Furthermore, we report higher rates of smell loss resolution in participants with partial compared to complete smell loss (67.4% vs. 54.5%, *p* = 0.032). This is compatible with Kosugi et al. [14] who reported that the full resolution from ‘partial loss of smell’ in COVID-19 positive patients takes place more frequently than that from ‘complete loss of smell’. Supportively, using an objective approach, Lechien et al. also found that higher baseline severity of smell loss, measured by ‘Sniffin-Sticks’ was strongly predictive of persistent smell loss [24]. Beltrán-Corbellini et al. compared smell loss recovery in 70 COVID-19 and 40 influenza participants [25]. 40% of COVID-19 positive participants reported full resolution after 7.4 ± 2.3 days and 16.7% reported partial resolution after 9.1 ± 3.6 days, whereas 100% of influenza participants fully recovered their sense of smell. The fact that participants with SARS-CoV-2 antibodies also had higher rates of complete anosmia, suggests a more severe spectrum of COVID-19 related smell loss compared to post-viral smell loss from other respiratory pathogens.

Interestingly, one of our key findings shows that parosmia was more common in the group of participants with unresolved smell loss and was also a predictor of non-remission in the logistic regression analysis, which is novel in COVID-19. Parosmia has been associated with post-viral smell loss prior to the COVID-19 pandemic [26]. A potential explanation for our finding may be that parosmia has been associated with decreased number and disordered regrowth of olfactory axons into existing neural circuits and a preponderance of immature neurons [27]. Comparatively, Liu et al. found that in 153 participants with post-infectious smell loss the presence of parosmia was associated with clinically significant recovery in suprathreshold olfactory function discrimination in patients receiving olfactory training [28]. Parosmia in the context of post-viral smell loss is associated with ongoing smell impairment, and although has been viewed as a sign of recovery, its role as a prognostic marker remains largely unclear. However, our data suggest that parosmia is a marker of poor prognosis in COVID-19. Similarly, our finding show that dysgeusia and experiencing taste sensations in the absence of eating and drinking were associated with lower reported taste loss resolution rates. Together, these findings suggest that distorted chemosensory perception is a risk factor for prolonged smell and/or taste loss and long-COVID.

With regard to full resolution of smell and/or taste loss, a significant sex difference was evident. Females had a lower full resolution rate of smell loss (51.4% vs. 72.8%; *p* < 0.001), taste loss (60.1% vs. 80.8%; *p* < 0.001) and combined smell and taste loss (45.1% vs. 69.6%; p < 0.001), compared to males, respectively. These findings are supported by several studies who also report the recovery of sense of smell was longer in females [10, 22]. Although Meini et al. found that the recovery rate did not differ significantly between males and females, they however, the mean recovery time from smell loss or taste loss was significantly longer for females than for males (26 vs. 14 days, *p* = 0.009), even though the mean age of males was significantly higher than that of females (66 vs. 57 years, *p* = 0.04) [21].

Notably, in our study, female participants who reported no/partial resolution of smell loss were significantly older (41.6 ± 11.7 years) than female participants who reported full resolution of smell loss (38.1 ± 11.3 years, *p* = 0.010). The same was also found to be true for taste loss (42.7 ± 12.5 vs. 37.1 ± 10.7, (*p* < 0.001) and combined smell and taste loss (42.8 ± 12.5 vs. 37.1 ± 11.0, p < 0.001). In contrast, Lee et al. reported that young age, particularly the age group of 20–39 years, showed a tendency to be associated with a longer persistence of anosmia [29]. Interestingly, oestradiol has been shown to increase olfactory epithelial cell density and to have a protective role against olfactory function decline [30]. Additionally, in animal models of neurogenerative diseases oestradiol replacement prevents olfactory dysfunction [31]. This study was not powered to estimate the effect of menopause-driven differences in this subgroup. Nevertheless, a link between prolonged or reduced recovery of smell function and a post-menopausal state appears plausible.

Loss of smell and taste have been demonstrated to persist beyond resolution of the infectious phase of COVID-19 [32]. In light of evidence from the clinical course of smell loss from other viruses, smell loss after COVID-19 could persist for two or more years [33, 34]. Arguably, our study follow-up window still only represents a relatively short-term observation and it is reasonable to predict that a number of participants will have further recovered their sense of smell and taste over additional weeks. Nevertheless, loss of smell and taste undoubtedly constitute a manifestation of long-COVID, which may result in significant psychological morbidity and adversely impact quality of life of the subset of patients with long-term unresolved smell and taste loss [8, 16, 35]. Given the limitations in accuracy and accessibility to testing during particularly the earlier phases of the pandemic, further patient populations may present with olfactory and gustatory impairment and features of long-COVID, indicative of previous COVID-19 infections. Of note, current proposed diagnostic criteria for long-COVID or chronic COVID-19 do not necessitate a positive test at time of symptom onset. The magnitude of the pandemic and the potential for SARS-CoV-2 to cause long-term smell and/or taste loss in relatively small cohorts therefore suggest that the overall prevalence of long-COVID with smell loss within the general population will be considerable [5, 9].

It will be vital to develop a better understanding of the pathophysiology causing smell loss as well as the physiological processes driving smell recovery, in order to facilitate development of effective treatments for smell loss. Angiotensin-converting enzyme-2 receptors (ACE-2) present on olfactory epithelial cells and neuropilin-1 receptors (NRP1), abundant on all olfactory cell lines, are known entry routes for SARS-CoV-2 and damage to these cells have been proposed as potential mechanisms for smell loss [36–38]. Viral entry via NRP1 could result in direct damage to olfactory sensory neurons [36]. Furthermore, the distribution of these two receptors may form a plausible explanation for the spectrum of smell loss, with mild and typically short-lived smell loss from ACE-2 invasion versus more longstanding smell loss via NRP1 mediated sensory neuronal damage, following COVID-19. Therapeutic strategies including olfactory rehabilitation and corticosteroids are already in use to aid recovery of smell function following COVID-19, with further therapeutic strategies in clinical trials [14, 15]. With emerging treatments for COVID-19 related anosmia and the high predicted prevalence of long covid with long-standing smell loss, identifying those at risk of long-COVID with smell loss will be key. We highlight female sex (and increasing age within female cohorts) as well as the presence of parosmia as key risk factors for a prolonged clinical course of COVID-19 related smell loss.

Finally, in this study, we addressed the limitation of using subjective assessment of smell by recruiting a subset of participants who underwent objective UPSIT testing. Our results show that objectively assessed smell function using UPSIT correlates well with perceived smell function in this population, which highlights the reliability of our subjective patient-reported data on smell function following COVID-19. This finding is in line with a study conducted in ambulatory COVID-19 patients using the 12-item Brief Smell Identification Test (BSIT) which also concluded that self-reported olfactory loss is a strong predictor of abnormal olfactory function [39]. Furthermore, this highlights that in this subsample, tested 21.6 ± 4.7 weeks since the onset of their symptoms, 20% of participants had ongoing loss of their sense of smell, who would meet diagnostic criteria for both long-COVID and chronic COVID-19.

### Limitations

The main limitation of this study remains the lack of a general population control group without loss of smell/taste. Our selection criteria enabled us to study acute smell and taste loss as presentations of COVID-19 as well as their resolution in people both with and without SARS-CoV-2 antibodies. Coupled with the web-based delivery of the study, this leads to susceptibility to a degree of selection and age bias, which required proficiency with computers and smartphones and may have resulted in under-representation of older adults. Furthermore, the majority of our participants were female; this may reflect previous findings that females are more likely to engage in research and also have a higher frequency of loss of smell and/or taste with COVID-19 than males. Through recruiting a subset of participants for objective testing we addressed the previous limitation of lack of objective testing, and demonstrated a strong correlation between perceived and assessed smell function.

## Conclusions

We followed up a community cohort of people who had reported acute loss of their sense of smell and/or taste and had undergone SARS-CoV-2 IgG/IgM antibody testing 4–6 weeks earlier in order to investigate the clinical course of smell and/or taste loss. We also aimed to identify factors that were associated with persistent smell loss at 4–6 weeks of follow-up. In line with existing literature, we can offer reassurance that smell and/or taste loss is a transient phenomenon in most SARS-CoV-2 cases. However, persistent smell and taste loss constitute a feature of long-COVID. The population of patients with longstanding smell and/or taste loss as a manifestation of long-COVID will continue to grow during and following the pandemic; given the impact of these symptoms on quality of life and safety, it will be imperative to devise support and treatment pathways. We identified the presence of parosmia and female sex as risk factors for persistent smell loss, as well as increasing age within the female sub-cohort. Similarly, female sex and increasing age as well as distorted taste perception were associated with persistent taste loss. Our findings highlight that female patients over the age of 40, who experience with a distorted perception of their sense of smell and/or taste are likely to benefit from therapeutic interventions to prevent persistent smell and/or taste loss and should be prioritized when targeted therapies for post-covid smell and taste loss become available.

## Supplementary Information

**Additional file 1.** Study questionnaires.

**Additional file 2.** Demographics of participants with positive and negative SARS-CoV-2 antibodies from entire study cohort.

## Data Availability

The datasets used and/or analysed during the current study are available from the corresponding author on reasonable request.

## Abbreviations

SARS-CoV-2: Severe Acute Respiratory Syndrome Coronavirus 2
COVID-19: Coronavirus disease 2019
IgG: Immunoglobulin G
IgM: Immunoglobulin M
UPSIT: University of Pennsylvania Smell Identification Test
ACE-2: Angiotensin-converting enzyme-2 receptors
NRP1: Neuropilin-1 receptors
BSIT: Brief Smell Identification Test

## References

[1] WHO. Coronavirus disease (COVID-19) pandemic 2020 [cited 2020 22 June]. Available from: https://www.who.int/emergencies/diseases/novel-coronavirus-2019.

[2] Menni C, Valdes AM, Freidin MB, Sudre CH, Nguyen LH, Drew DA, et al. Real-time tracking of self-reported symptoms to predict potential COVID-19. Nat Med. 2020. 10.1038/s41591-020-0916-2 PMC7751267 32393804

[3] Hopkins C, Surda P, Kumar N. Presentation of new onset anosmia during the COVID-19 pandemic. Rhinology. 2020. 10.4193/Rhin20.116 32277751

[4] Makaronidis J, Mok J, Balogun N, Magee CG, Omar RZ, Carnemolla A (2020). Seroprevalence of SARS-CoV-2 antibodies in people with an acute loss in their sense of smell and/or taste in a community-based population in London, UK: an observational cohort study. PLoS Med.

[5] Sudre CH, Murray B, Varsavsky T, Graham MS, Penfold RS, Bowyer RC (2020). Attributes and predictors of Long-COVID: analysis of COVID cases and their symptoms collected by the Covid Symptoms Study App. medRxiv.

[6] Greenhalgh T, Knight M, A’Court C, Buxton M, Husain L (2020). Management of post-acute covid-19 in primary care. BMJ..

[7] NICE. COVID-19 rapid guideline: managing the long-term effects of COVID-19 nice.org.uk: NICE Guidance; 2021 [Available from: https://www.nice.org.uk/guidance/NG188. 33555768

[8] ONS. Office for National Statistics. The prevalence of long COVID symptoms and COVID-19 complications. 2020 [Available from: https://www.ons.gov.uk/news/statementsandletters/theprevalenceoflongcovidsymptomsandcovid19complications.

[9] Soler ZM, Patel ZM, Turner JH, Holbrook EH (2020). A primer on viral-associated olfactory loss in the era of COVID-19. Int Forum Allergy Rhinol..

[10] Suzuki M, Saito K, Min WP, Vladau C, Toida K, Itoh H (2007). Identification of viruses in patients with postviral olfactory dysfunction. Laryngoscope.

[11] Boscolo-Rizzo P, Borsetto D, Fabbris C, Spinato G, Frezza D, Menegaldo A, et al. Evolution of altered sense of smell or taste in patients with mildly symptomatic COVID-19. JAMA Otolaryngol Head Neck Surg. 2020. 10.1001/jamaoto.2020.1379 PMC7333173 32614442

[12] Chary E, Carsuzaa F, Trijolet JP, Capitaine AL, Roncato-Saberan M, Fouet K (2020). Prevalence and recovery from olfactory and gustatory dysfunctions in Covid-19 infection: a prospective multicenter study. Am J Rhinol Allergy.

[13] Otte MS, Klussmann JP, Luers JC (2020). Persisting olfactory dysfunction in patients after recovering from COVID-19. J Inf Secur.

[14] Whitcroft KL, Hummel T. Olfactory dysfunction in COVID-19: diagnosis and management. JAMA. 2020. 10.1001/jama.2020.8391 32432682

[15] ClinicalTrials.gov. Coronavirus Smell Therapy for Anosmia Recovery 2021 [Available from: https://www.clinicaltrials.gov/ct2/show/NCT04422275.

[16] Croy I, Nordin S, Hummel T (2014). Olfactory disorders and quality of life--an updated review. Chem Senses.

[17] Elabscience. COVID-19 IgG and IgM [Available from: https://www.elabscience.com/p-covid_19_igg_igm_rapid_test-375335.html.

[18] Doty RL, Shaman P, Kimmelman CP, Dann MS (1984). University of Pennsylvania Smell Identification Test: a rapid quantitative olfactory function test for the clinic. Laryngoscope.

[19] Wajnberg A, Amanat F, Firpo A, Altman DR, Bailey MJ, Mansour M (2020). Robust neutralizing antibodies to SARS-CoV-2 infection persist for months. Science (New York, NY).

[20] Rodda LB, Netland J, Shehata L, Pruner KB, Morawski PA, Thouvenel CD (2021). Functional SARS-CoV-2-Specific Immune Memory Persists after Mild COVID-19. Cell.

[21] Meini S, Suardi LR, Busoni M, Roberts AT, Fortini A (2020). Olfactory and gustatory dysfunctions in 100 patients hospitalized for COVID-19: sex differences and recovery time in real-life. Eur Arch Otorhinolaryngol.

[22] Dell'Era V, Farri F, Garzaro G, Gatto M, Aluffi Valletti P, Garzaro M (2020). Smell and taste disorders during COVID-19 outbreak: cross-sectional study on 355 patients. Head Neck.

[23] Vaira LA, Hopkins C, Petrocelli M, Lechien JR, Chiesa-Estomba CM, Salzano G (2020). Smell and taste recovery in coronavirus disease 2019 patients: a 60-day objective and prospective study. J Laryngol Otol.

[24] Lechien JR, Journe F, Hans S, Chiesa-Estomba CM, Mustin V, Beckers E (2020). Severity of Anosmia as an Early Symptom of COVID-19 Infection May Predict Lasting Loss of Smell. Front Med (Lausanne).

[25] Beltrán-Corbellini Á, Chico-García JL, Martínez-Poles J, Rodríguez-Jorge F, Natera-Villalba E, Gómez-Corral J (2020). Acute-onset smell and taste disorders in the context of COVID-19: a pilot multicentre polymerase chain reaction based case-control study. Eur J Neurol.

[26] Reden J, Maroldt H, Fritz A, Zahnert T, Hummel T (2007). A study on the prognostic significance of qualitative olfactory dysfunction. Eur Arch Otorhinolaryngol.

[27] Leopold DA, Loehrl TA, Schwob JE (2002). Long-term follow-up of surgically treated phantosmia. Arch Otolaryngol Head Neck Surg.

[28] Liu DT, Sabha M, Damm M, Philpott C, Oleszkiewicz A, Hähner A, et al. Parosmia is Associated with Relevant Olfactory Recovery After Olfactory Training. The Laryngoscope. 2020. 10.1002/lary.29277 33210732

[29] Lee Y, Min P, Lee S, Kim SW (2020). Prevalence and duration of acute loss of smell or taste in COVID-19 patients. J Korean Med Sci.

[30] Dhong HJ, Chung SK, Doty RL (1999). Estrogen protects against 3-methylindole-induced olfactory loss. Brain Res.

[31] Nathan BP, Tonsor M, Struble RG (2010). Acute responses to estradiol replacement in the olfactory system of apoE-deficient and wild-type mice. Brain Res.

[32] Yan CH, Prajapati DP, Ritter ML, DeConde AS (2020). Persistent smell loss following undetectable SARS-CoV-2. Otolaryngol Head Neck Surg.

[33] Cavazzana A, Larsson M, Münch M, Hähner A, Hummel T (2018). Postinfectious olfactory loss: a retrospective study on 791 patients. Laryngoscope.

[34] Hwang CS (2006). Olfactory neuropathy in severe acute respiratory syndrome: report of a case. Acta Neurol Taiwanica.

[35] Erskine SE, Philpott CM (2020). An unmet need: patients with smell and taste disorders. Clin Otolaryngol.

[36] Hopkins C, Lechien JR, Saussez S. More that ACE2? NRP1 may play a central role in the underlying pathophysiological mechanism of olfactory dysfunction in COVID-19 and its association with enhanced survival. Med Hypotheses. 2020;110406. 10.1016/j.mehy.2020.110406 PMC7678428 33246692

[37] Bryche B, St Albin A, Murri S, Lacôte S, Pulido C, Ar Gouilh M (2020). Massive transient damage of the olfactory epithelium associated with infection of sustentacular cells by SARS-CoV-2 in golden Syrian hamsters. Brain Behav Immun.

[38] Hoffmann M, Kleine-Weber H, Schroeder S, Krüger N, Herrler T, Erichsen S (2020). SARS-CoV-2 Cell Entry Depends on ACE2 and TMPRSS2 and Is Blocked by a Clinically Proven Protease Inhibitor. Cell.

[39] Prajapati DP, Shahrvini B, MacDonald BV, Crawford KL, Lechner M, DeConde AS, et al. Association of subjective olfactory dysfunction and 12-item odor identification testing in ambulatory COVID-19 patients. Int Forum Allergy Rhinol. 2020. 10.1002/alr.22688 32964657

