# Supplementary material for: Distorted chemosensory perception and female sex associate with persistent smell and/or taste loss in people with SARS-CoV-2 antibodies: a community based cohort study investigating clinical course and resolution of acute smell and/or taste loss in people with and without SARS-CoV-2 antibodies in London, UK
Source: BMC Infect Dis. 2021 Feb 25;21:221. doi: 10.1186/s12879-021-05927-w (PMC7905973; doi:10.1186/s12879-021-05927-w)
Supplement: Supplementary file 1 — Additional file 1. Study questionnaires. [file 12879_2021_5927_MOESM1_ESM.docx]

**Supporting Information: Part 1: Participant demographics questions**

|  | Question | Answer field |
| --- | --- | --- |
| 1 | **Date of birth** | DD/MM/YYYY |
| 2 | **Sex** | Female  Male  Other |
| 3 | **Ethnicity** | White  Black  Asian  Mixed  Other  I do not wish to disclose |
| 4 | **Have you ever smoked?** | Current smoker  Ex-smoker  Never smoked |

**Supporting Information: Part 2: Participant baseline symptom questionnaire**

|  | Question | Answer field | |
| --- | --- | --- | --- |
| 1 | **Have you noticed any changes in your sense of smell?** | Yes  No | |
| 1.2 | **How would you describe the change in your sense of smell?** | - I can/could no longer smell any odours that I used to be able to smell - I can/could no longer smell some odours I used to be able to smell  Describe (optional) | |
| 1.3 | **Have you noticed that odours smell different than they used to?** | No  Yes  If yes describe (optional) | |
| 2 | **Have you noticed any changes in your sense of taste?** | Yes  No | |
| 2.1 | **How would you describe the changes in your sense of taste?** | - I can/could no longer taste any foods or drinks - My ability to taste food or drinks is/was reduced Describe (optional) | |
| 2.2 | **Have you noticed that foods and drinks taste differently?** | Yes  No  If yes describe (optional) | |
| 2.3 | **Have you experienced any unusual tastes while not eating or drinking?** | Yes  No  If yes describe | |
| 3 | **Have you experienced any of these symptoms in the last 4 weeks?** | Cough | Yes/No |
|  |  | Fever (37.8◦C or more) |  |
|  |  | Shortness of breath |  |
|  |  | Headache |  |
|  |  | Sore throat |  |
|  |  | Hoarse voice |  |
|  |  | Chest pain/tightness |  |
|  |  | Abdominal pain |  |
|  |  | Diarrhoea |  |
|  |  | Vomiting |  |
|  |  | Confusion, disorientation or drowsiness |  |
|  |  | Muscle/joint aches |  |
| 4 | **Has/was your appetite for food decreased?** | Yes  No | |
| 5 | **Have you had a test for COVID-19?** | Yes  No  If Yes: positive negative Type of test: swab test blood test  Location of test | |

**Supporting Information: Part 3: Participant follow-up symptom questionnaire**

|  | Question | Answer field | |
| --- | --- | --- | --- |
|  | Participant number |  | |
| 1 | **Did the changes in your sense of smell resolve?** | Resolved fully Date:  Resolved partially  Did not resolve | |
| 2 | **Did the changes in your sense of taste resolve?** | Resolved fully Date:  Resolved partially  Did not resolve | |
| 3 | **Did your other symptoms resolve?**  If yes please enter the date these resolved | Cough | Resolved? Yes/No |
|  |  | Fever (37.8◦C or more) |  |
|  |  | Shortness of breath |  |
|  |  | Chest pain/tightness |  |
|  |  | Headache |  |
|  |  | Sore throat |  |
|  |  | Hoarse voice |  |
|  |  | Abdominal pain |  |
|  |  | Diarrhoea |  |
|  |  | Vomiting |  |
|  |  | Confusion, disorientation or drowsiness |  |
|  |  | Muscle/joint aches |  |
| 4 | **Have you developed any of the following symptoms in the last 4 weeks?** | Cough | Yes/No |
|  |  | Fever (37.8◦C or more) |  |
|  |  | Shortness of breath |  |
|  |  | Chest pain/tightness |  |
|  |  | Headache |  |
|  |  | Sore throat |  |
|  |  | Hoarse voice |  |
|  |  | Abdominal pain |  |
|  |  | Diarrhoea |  |
|  |  | Vomiting |  |
|  |  | Confusion, disorientation or drowsiness |  |
|  |  | Muscle/joint aches |  |
| 5 | **Have you attended/been admitted to hospital due to COVID-19?** | Yes  No | |
