# Supplementary material for: Distorted chemosensory perception and female sex associate with persistent smell and/or taste loss in people with SARS-CoV-2 antibodies: a community based cohort study investigating clinical course and resolution of acute smell and/or taste loss in people with and without SARS-CoV-2 antibodies in London, UK
Source: BMC Infect Dis. 2021 Feb 25;21:221. doi: 10.1186/s12879-021-05927-w (PMC7905973; doi:10.1186/s12879-021-05927-w)
Supplement: Supplementary file 2 — Additional file 2. Demographics of participants with positive and negative SARS-CoV-2 antibodies from entire study cohort. [file 12879_2021_5927_MOESM2_ESM.docx]

**Additional File 2**

**Demographics of participants with positive and negative SARS-CoV-2 antibodies**

| **Demographics** | **SARS-CoV-2 antibody positive (N=443)** | **SARS-CoV-2 antibody negative (N=126)** | **p-value** |
| --- | --- | --- | --- |
| **SARS-CoV-2 IgG/IgM test** | 77.9%  (n=443) | 22.1%  (n=126) |  |
| **Gender** |  |  |  |
| Female | 70.2%  (n=311) | 64.3%  (n=81) | 0.205 |
| Male | 29.3%  (n=130) | 35.7%  (n=45) | 0.172 |
| Other | 0.5%  (n=2) | n=0 | 0.450 |
| **Age (years)** | 39.4 (±11.9) | 40.5 (±12.6) | 0.391 |
| **Ethnicity** |  | | |
| White | 81.5%  (n=361) | 78.6%  (n=99) | 0.728 |
| Mixed/Multiple Ethnicities | 5.9%  (n=26) | 7.1%  (n=9) | 0.563 |
| Asian/Asian British | 5.9%  (n=26) | 5.6%  (n=7) | 0.931 |
| Black/African/Caribbean/Black British | 2.3%  (n=10) | 2.4%  (n=3) | 0.910 |
| ‘Other’ | 4.1%  (n=18) | 4%  (n=5) | 0.992 |
| **Smoking status** |  | | |
| Current/ Ex-smoker | 41.8%  (n=185) | 48.4%  (n=61) | 0.246 |
| Never smoked | 58.2%  (n=258) | 51.6%  (n=65) |  |

*Figures presented as % with total number (n). SARS-CoV-2, severe acute respiratory syndrome coronavirus 2.*
